# KIR and HLA-C Interactions Promote Differential Dendritic Cell Maturation and Is a Major Determinant of Graft Failure following Kidney Transplantation

**DOI:** 10.1371/journal.pone.0023631

**Published:** 2011-08-31

**Authors:** Raj Hanvesakul, Chandrashekhar Kubal, Jason Moore, Desley Neil, Mark Cook, Simon Ball, David Briggs, Paul Moss, Paul Cockwell

**Affiliations:** 1 Renal Institute of Birmingham, University Hospital Birmingham, Birmingham, United Kingdom; 2 Department of Pathology, University Hospital Birmingham, Birmingham, United Kingdom; 3 Department of Haematology, University Hospital Birmingham, Birmingham, United Kingdom; 4 Department of Histocompatibility & Immunogenetics, National Blood Service, Vincent Drive, Birmingham, United Kingdom; 5 Division of Cancer Studies, University of Birmingham, Birmingham, United Kingdom; 6 Division of Immunity and Infection, University of Birmingham, Birmingham, United Kingdom; Dana-Farber Cancer Institute, United States of America

## Abstract

**Background:**

HLA-C is an important ligand for killer immunoglobulin like receptors (KIR) that regulate natural killer (NK) cell function. Based on KIR specificity HLA-C molecules are allocated into two groups, HLA-C1 or HLA-C2; HLA-C2 is more inhibiting to NK cell function than HLA-C1. We studied the clinical importance of HLA-C genotypes on the long-term graft survival of 760 kidney transplants performed at our centre utilising a population based genetic study and cell culture model to define putative mechanisms.

**Methods and Findings:**

Genotyping was performed using conventional DNA PCR techniques and correlations made to clinical outcomes. We found that transplant recipients with HLA-C2 had significantly better long-term graft survival than transplant recipients with HLA-C1 (66% versus 44% at 10 years, log-rank p = 0.002, HR = 1.51, 95%CI = 1.16–1.97). In in-vitro NK and dendritic cell (DC) co-culture model we made several key observations that correlated with the population based genetic study. We observed that donor derived NK cells, on activation with IL-15, promoted differential HLA-C genotype dependent DC maturation. In NK-DC co-culture, the possession of HLA-C2 by DC was associated with anti-inflammatory cytokine production (IL-1RA/IL-6), diminished DC maturation (CD86, HLA-DR), and absent CCR7 expression. Conversely, possession of HLA-C1 by DC was associated with pro-inflammatory cytokine synthesis (TNF-α, IL-12p40/p70), enhanced DC maturation and up-regulation of CCR7 expression. By immunohistochemistry the presence of donor NK cells was confirmed in pre-transplant kidneys.

**Conclusions:**

We propose that after kidney transplantation IL-15 activated donor derived NK cells interact with recipient DC with less activation of indirect allo-reactivity in HLA-C2 positive recipients than HLA-C1 positive recipients; this has implications for long-term graft survival. Early events following kidney transplantation involving NK-DC interaction via KIR and HLA-C immune synapse may have a central role in long-term kidney transplant outcomes.

## Introduction

Kidney transplantation is the standard of care for many people with end stage kidney disease [1]. However, whilst acute rejection rates and early graft loss have improved substantially over the past four decades, progressive chronic allograft injury (CAI) remains a very common cause of late graft loss [2], [3]. A major component of CAI is orchestrated by the adaptive components of the immune system including dendritic cells (DC), T cells and B cells [4]–[8]. Our knowledge of the link between innate and adaptive immunity in CAI including the contribution of NK cells is incomplete. This is an important shortfall, as NK cells have a central role in modulating the development of the adaptive response through interactions with HLA-C molecules on target cells [9], [10]. HLA-C molecules act as ligands for NK cell expressed inhibitory killer immunoglobulin-like receptors (KIR), with subsequent modulation of NK cell function. HLA-C molecules are allocated into two groups based on their KIR specificity: (i) HLA-C group 1 (C1) specific for KIR receptor 2DL2/3; (ii) HLA-C group 2 (C2) specific for KIR receptor 2DL1 [10]. Differential KIR and HLA expression appear to influence clinical outcomes in various diseases including cervical neoplasia [11], pre-eclampsia [12], antiviral immune response [13], hepatitis C [14] and liver transplantation [15]. As the co-expression of KIR 2DL1 and 2DL3 on NK cells occurs in greater than 90% of the population, the major determinant of NK cell inhibition is the differential expression of HLA-C ligands. Functional studies performed by Ahlenstiel and colleagues (2008) investigating antiviral responses in-vitro showed diminished degranulation and cytokine production by NK cells in HLA-C2 compared with HLA-C1 targets. They proposed that NK cell inhibition through interactions between KIR*2DL3* and HLA-C1 is weaker than inhibition conferred through KIR*2DL1*/HLA-C2 interactions [13].

In liver transplantation, we found that HLA-C2 expression by the allograft was protective against the development of chronic rejection, graft cirrhosis and late graft loss [15]. There is limited data on the effect of KIR and KIR ligands on clinical outcomes following kidney transplantation. In a study of 224 kidney transplant recipients, Kunert and colleagues (2007) showed that expression of a HLA-C2 homozygous genotype by the allograft was associated with a reduced incidence of acute rejection [16]. Nowak and colleagues (2010) genotyped 285 recipients of kidney transplants and demonstrated that the presence of HLA-C2 and absence of KIR*2DS5* in the recipient correlated with increased episodes of acute allograft rejection. Conversely they found that the presence of both HLA-C1 and KIR*2DS5* in the recipient was protective against acute allograft rejection [17]. In a study comprising of 2,757 kidney transplants, Tran and colleagues (2005) investigated the impact of KIR ligand matching on graft survival. Whilst they showed no correlation between KIR ligand matching and graft survival they did not specifically investigate the relationship between differential HLA-C expression and graft outcomes [18].

The predominant subgroup of NK cells (>95% of peripheral blood NK cells) are CD56^dim^CD16^+^ and possess KIR receptors (95%) [19], [20]. In addition to their role in the elimination of tumour and virus transformed cells, they also interact with DC. This interaction is contact dependent and bidirectional, involving multiple cytokine synthesis including IFN-γ, TNF-α, IL-12, IL-15, IL-18 and HMGB1 [21]–[25]. During NK-DC co-culture, NKp30 engagement triggers intracellular mechanisms that are further modulated by KIR and HLA-C interactions [26], [27]. Dendritic cells that are matured during NK-DC crosstalk are potent T-cell primers [28] and promote Th1 polarisation [29].

Following kidney transplantation, the allograft undergoes significant ischaemia reperfusion injury (IRI) with release of pro-inflammatory cytokines [30] including IL-15 [31]–[33] and recruitment of recipient monocytes and DCs [34]. Passenger leukocytes transferred in the allograft from donor to recipient augment the allo-immune response. Allogeneic donor derived NK cells transferred by this process may be a predominant activator of recipient DCs. Furthermore DC maturation supersedes cytolysis during IRI as products of cellular damage trigger TLR4 expressed on DC [30]. Even in the presence of conventional immunotherapy, IL-15 will promote NK-DC crosstalk, leading to accelerated maturation of DC with allo-antigen presenting capacity.

In this study, we investigated the influence of HLA-C genotype on graft survival. We found that recipients with HLA-C2 had significantly better long-term graft survival after kidney transplantation than those without HLA-C2 (i.e. HLA-C1 homozygotes). Based on these observations we hypothesised that the interaction between allogeneic donor derived NK cells transferred in the allograft at time of transplantation and recipient DC are differentially modulated by the engagement of donor KIR and recipient HLA-C on DC. This hypothesis was based on the premise that as HLA-C2 is more inhibiting to NK cell function than HLA-C1, DC expressing HLA-C2 should undergo less maturation than those expressing HLA-C1, with a subsequent impact on T-cell priming. This was investigated by an NK-DC co-culture model controlled for confounding factors by utilising: (i) NK cells from donors heterozygous for HLA-C (*C1/C2*), negative for HLA-Bw4, haplotype AA with one activating KIR*2DS4* and with 3 inhibitory KIR*2DL1*, *2DL3*, and *3DL1*; (ii) DC from donors negative for HLA-Bw4 and either homozygous for HLA-C1 or HLA-C2. This process of selection excluded the potential confounding effects of HLA-Bw4 through interactions with 3DL1 and allowed direct comparisons to be made between HLA-C1 homozygous and HLA-C2 homozygous DCs. Under these conditions, allogeneic NK-DC co-cultures facilitated an assessment of the mechanisms of the association of HLA-C genotype with graft survival.

## Materials and Methods

### Ethics Statement

Approvals for all parts of this study were granted by the South Birmingham Research Ethics Committee. Informed consent was not required as data were analyzed anonymously. The ethics committee specifically waived the need for consent.

### Population Genetics

Nine hundred and fifty adult kidney transplant recipients were treated at the Queen Elizabeth Hospital, Birmingham between 1996 and 2004. DNA was available for a total of 890 kidney donors and 760 transplant recipients. The final study population therefore comprised 760 renal transplant pairs all with complete long-term follow-up; 640 were deceased donor transplants and 120 were live donor transplants; 652 recipients were first time kidney transplants, 86 were second and 22 were third transplant.

By polymerase chain reaction sequence specific primer (PCR-SSP) technique, donor and recipient DNA were genotyped for the presence of the three major KIR ligand groups, HLA-C1, HLA-C2 and HLA-Bw4. These were assigned directly by using specific oligonucleotide primers to type the codon corresponding to amino acid 80 for HLA-C and codons 80–83 for HLA-Bw4. Similarly, KIR genotyping was performed for inhibitory and activating KIR using PCR-SSP with primers based on those published by Uhrberg [35]; Four inhibitory KIRs (2DL1, 2DL2, 2DL3 and 3DL1) and seven activating KIRs (2DS1, 2DS2, 2DS3, 2DS4, 2DS4v, 2DS5, and 3DS1) were analysed. The clinical outcome data comprised biopsy proven acute rejection, graft survival and patient survival. Graft loss was defined as graft failure requiring dialysis (n = 163). Both death non-censored and death censored graft survival were calculated. For death non-censored graft survival, patient death with a functioning graft was also defined as graft failure and therefore included as graft loss (n = 219). Causes of graft loss and patient death are listed in Table 1 and 2. Patient death (n = 56, Table 2) from all causes was included in the analysis of patient survival. Preliminary power calculations were performed for this study and based on the assumption that 10-year graft survival for kidney transplants at our centre is 65%, then for 760 cases (assuming 40% are HLA-C1 homozygous and the remaining 60% are in the comparator group i.e. the presence of HLA-C2 allele therefore the heterozygous and HLA-C2 homozygous genotype combined) this study size has a 90% power to detect a hazard ratio of 1.49 for graft failure.

**Table 1:** Causes of graft loss following kidney transplantation.

Cause of graft loss (n = 163)	Incidence
Chronic allograft injury	124 (76%)
Vascular thrombosis	24 (14.7%)
Acute rejection	7 (4.3%)
Recurrent of primary renal disease	6 (3.7%)
Infection of allograft	2 (1.3%)

**Table 2:** Causes of patient death following kidney transplantation.

Cause of patient death (n = 56)	Incidence
Cardiovascular disease	20 (35.7%)
Post-transplant lymphoproliferative disease	10 (17.9%)
Septicaemia	8 (14.3%)
Respiratory failure	6 (10.7%)
Cerebrovascular event	6 (10.7%)
Other malignant disease	6 (10.7%)

### Immunohistochemistry

Pre-transplant kidney (n = 5) biopsies were available and assessed for the presence of donor derived NK cells. Biopsies were taken prior to transplantation, and fixed in formalin. The method used for the development of slides include dewaxing and antigen retrieval obtained by W-cap system (Bio-Optica) and staining with a mouse monoclonal anti-CD56 antibody (IgG2b Novocastra) used at a dilution of 1∶50 and visualised with the EnVision detection system (DAKO). Negative controls were performed using corresponding isotype antibody staining. Cell counts (degree of infiltration) were performed using light microscopy and counting 10 randomly selected high power fields at a magnification of 400× (area = 0.17 mm^2^).

### In-vitro NK-DC co-culture

In order to analyse the influence of DC HLA-C genotype on NK-cell mediated maturation of DC, we developed a co-culture model to exclude the known confounding factors detailed in the introduction.

Whole blood samples were obtained from healthy laboratory donors for the isolation of purified and enriched NK and DC cell populations from PBMC using magnetic cell separation. The development of an NK-DC co-culture system required two independent steps. Firstly, DC were prepared using a pre-optimised magnetic cell sorting kit (Miltenyi Biotec, Surrey, UK) for the extraction of CD14^+^ monocytes from PBMC. Following isolation, CD14^+^ monocytes were treated with 100 ng/ml of GM-CSF (Peprotech, London, UK) and 1000 U/ml of IL-4 (Peprotech, London, UK) in RPMI containing 5% autologous serum and maintained in a 5% CO_2_ cell culture incubator at 37°C for 5 days. This altered their phenotype to that of immature dendritic cells (iDC) with a population of >90% CD14^−^CD1a^+^ iDC cells, comparable with the published literature [21]–[23]. On day 5, NK cells were isolated from PBMC using a pre-optimised magnetic cell sorting kit (Miltenyi Biotec, Surrey, UK) for the extraction of CD56^+^CD16^+^ NK cell. Purities of >98% were attained consistent with published literature [21]–[23]. Allogeneic NK and iDC cells were then co-cultured in the presence or absence of 1 ng/ml IL-15 (Peprotech, London, UK) in RPMI containing 10% foetal calf serum (Sigma-Aldrich, Dorset, UK) for 48 hours in a 48 well plate maintained in a 5% CO_2_ cell culture incubator at 37°C. Co-culture cell ratios of 1∶1 and 1∶5 (NK∶DC) were then studied as these ratios are most favourable for DC maturation [21] and should be consistent with the in-vivo relationship. Typically (at 1∶1) each well contained 0.25×10^6^ of each cell type in a final media volume of 500 µl. The control wells had either DC or NK cells in isolation. Trans-well experiments were performed using the same conditions, but in the presence or absence of a 0.4 µm insert (Costar, Fisher Scientific, UK) to separate NK cells and DC. At the end of co-culture DC were tested for the expression of the maturation markers CD86 (PE, 2331, BD Bioscience) and HLA-DR (FITC, G46-6, BD Bioscience) and the chemokine receptor CCR7 (PE, 150503, R&D Systems). The DC gate on flow cytometry was defined by a combination of scatter plot and CD56 staining to identify NK cells. The Δ Mean Fluorescence Intensity (Δ MFI) per experiment for CD86, HLA-DR and CCR7 was calculated as the difference in MFI for DC in co-culture with NK cells versus DC in isolation (background maturation). Corresponding isotype controls (BD Bioscience) were used. Cells were analysed using a FACSCalibur flow cytometer (Becton Dickinson) with Winmdi 2.9 software (Scripps Research), acquiring information from a total of 5,000 gated cells.

Co-culture supernatants were also collected and tested for the presence of cytokines by multiplex assays [Cytokine 25-plex AB Bead Kit, Human (BioSource™), from Invitrogen; data analysed using Luminex®100™ Analyser].

Comparison of means for ΔMFI and supernatant cytokine synthesis was performed to compare responses for DC with HLA-C1 genotype (n = 4) versus DC with HLA-C2 genotype (n = 4). The NK and DC used in each experiment came from different lab donors thus making these allogeneic interactions.

### Statistical analysis

Actuarial data were analysed using the Kaplan-Meier method and the log-rank test. Cox regression models for multivariable analysis were used to identify independent factors contributing to long-term graft loss. Mann-Whitney U test (Exact 2-tailed significance) was used for comparing means of ΔMFI and supernatant cytokines synthesis. All other correlations for variables and comparison of means were performed using Kendall's tau-b and ANOVA tests respectively. Probability (p) values of less than 0.05 were considered significant for our analysis. All statistics were performed using SPSS 14.0.

## Results

### HLA-C2 positive kidney transplant recipients have significantly better long-term graft survival

Kaplan Meier survival analysis was performed for individual KIR, KIR haplotypes and HLA-C/Bw genotypes for both donor and recipient. Recipient HLA-C genotype was the only factor found to influence survival outcomes and therefore tested further. Frequency of genotypes was consistent with previous published reports. Donor frequency for individual genes: *2DL1* 98%, *2DL2* 46%, *2DL3* 92%, *3DL1* 94%, *2DS1* 40%, *2DS2* 48%, *2DS3* 30%, *2DS4* 96%, *2DS5* 31%, *3DS1* 37%, HLA-C1 87%, HLA-C2 62%, HLA-Bw4 35% and Haplotype AA 32%. Recipient frequency for individual genes: *2DL1* 95%, *2DL2* 36%, *2DL3* 97%, *3DL1* 89%, *2DS1* 28%, *2DS2* 44%, *2DS3* 28%, *2DS4* 83%, *2DS5* 33%, *3DS1* 34%, HLA-C1 87%, HLA-C2 57%, HLA-Bw4 38% and Haplotype AA 31%.

**Figure 1:**
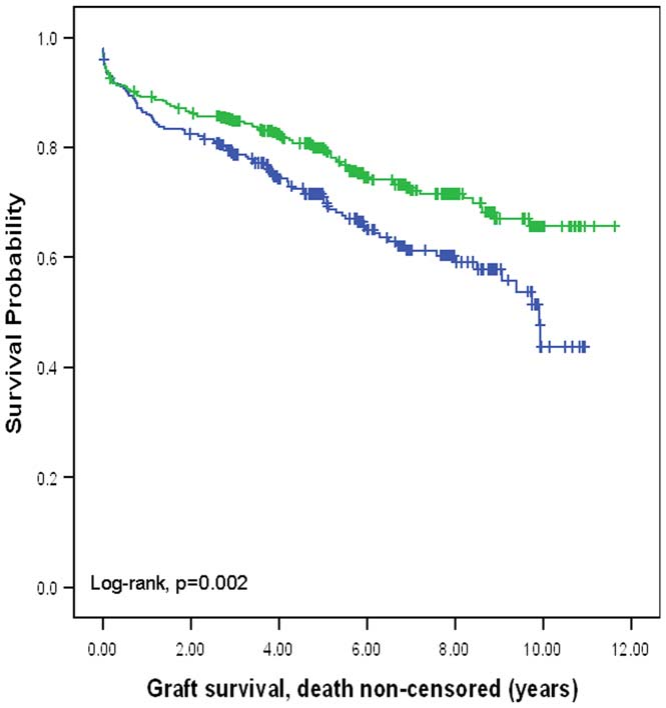
Kaplan Meier survival curve showing death non-censored graft survival for the presence or absence of HLA-C2 allele in the recipient. The presence of an HLA-C2 allele in the recipient was associated with a significant improvement in death non-censored graft survival (10-year survival: 65.7% versus 43.8%).

**Figure 2:**
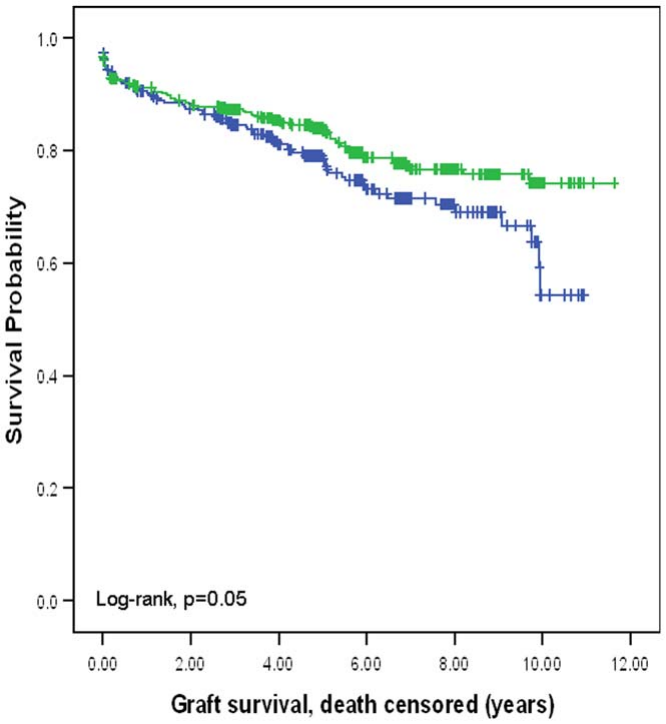
Kaplan Meier survival curve showing death censored graft survival for the presence or absence of HLA-C2 allele in the recipient. The presence of an HLA-C2 allele in the recipient was associated with a significant improvement in death censored graft survival (10-year survival: 74.2% versus 54.3%).

**Figure 3:**
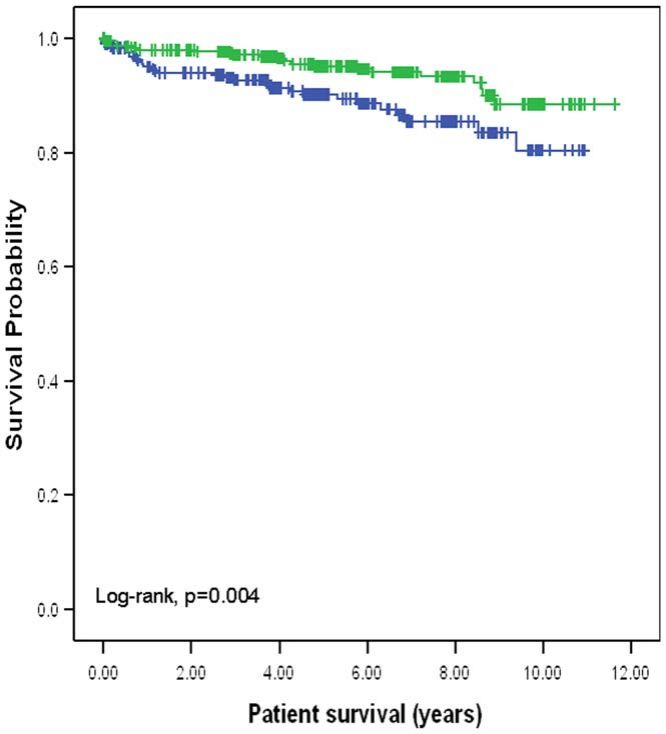
Kaplan Meier survival curve showing patient survival for the presence or absence of HLA-C2 allele in the recipient. The presence of an HLA-C2 allele in the recipient was associated with a significant improvement in patient survival (10-year survival: 88.5% versus 80.4%).

In contrast to liver transplantation, donor HLA-C genotype did not have impact on survival outcomes in kidney transplantation. However, recipient HLA-C genotype did have a major impact on outcomes 10-year death non-censored graft survival was significantly better for recipients with HLA-C2 compared with recipients without HLA-C2 (65.7%. versus 43.8%, p = 0.002; hazard ratio: 1.51, 95% CI: 1.16–1.97) (figure 1). Ten-year death censored graft survival was also significantly better for recipients with HLA-C2 compared with recipients without HLA-C2 (74.2% versus 54.3%, p = 0.05; hazard ratio: 1.36, 95% CI: 1.02–1.85) (figure 2). Ten-year patient survival was significantly better for recipients with HLA-C2 compared with recipients without HLA-C2 (88.5% versus 80.4%, p = 0.004; hazard ratio: 2.12, 95% CI: 1.26–3.58) (figure 3). HLA-C2 homozygous and heterozygous genotypes had comparable survival outcomes, and both groups were superior to HLA-C1 homozygotes (HLA-C2/C2 versus HLA-C1/C2; ten-year death non-censored graft survival 69.1% versus 64.1%, log-rank p = 0.66; ten-year death censored survival 80.5% versus 72.5%, log-rank p = 0.09; ten-year patient survival 88% versus 87%, log-rank p = 0.78). Re-transplants were not excluded from this analysis because analysing re-transplants only demonstrated similar survival benefit seen for recipients with HLA-C2 alleles. For re-transplants 10-year death non-censored graft survival was significantly better for recipients with HLA-C2 compared with recipients without HLA-C2 (60% versus 17.4%, p = 0.02; hazard ration: 2.17, 95% CI: 1.11–4.25). Ten-year death censored graft survival was also significantly better for recipients with HLA-C2 compared with recipients without HLA-C2 (72% versus 33%, p = 0.05; hazard ratio: 1.3, 95% CI: 1.23–1.7). Ten-year patient survival was significantly better for recipients with HLA-C2 compared with recipients without HLA-C2 (77% versus 52%, p = 0.015; hazard ratio: 3.7, 95% CI: 1.19–11.89) (figures not shown). This observation further supports a recipient derived effect. Donor and recipient HLA-Bw4, KIR haplotypes or KIR genotypes did not influence clinical outcomes following kidney transplantation. Furthermore KIR ligand matching did not influence clinical outcomes consistent with previous observations [17].

Multivariable analysis was performed for variables that may influence clinical outcomes in kidney transplantation (Table 3&4). This confirmed the association of the recipient HLA-C2 allele with improved graft survival for both death non-censored and death censored graft survival. Interestingly, in the multivariable analysis, recipient HLA-C2 allele was not significantly associated with improved patient survival. Other than 4 HLA antigen mismatches we did not observe the relationship between HLA mismatch and graft survival. Whilst HLA mismatching is known to influence graft survival our study was not designed to detect these as the number of cases per HLA mismatch were small.

**Table 3:** Demographics and distribution of parameters that are known to affect graft and patient survival after kidney transplantation.

Demographics	Absence of HLA-C2(n = 303)	Presence of HLA-C2(n = 457)	P value
Recipient age	43.0 (CI 41.2–44.8)	42.3 (CI 40.9–43.6)	0.49*
Donor age	43.5 (CI 41.6–45.4)	43.5 (CI 42.1–44.9)	0.96*
Recipient sex M∶F	191∶112	278∶179	0.55+
Donor sex M∶F	180∶123	236∶221	0.03+
Seropositive CMV in recipient	169	240	0.43+
Seropositive CMV in donor	150	212	0.41+
Type of transplant; Deceased donor: Live donor	258∶45	382∶75	0.60+
Number of transplant			
1	256	398	0.26+
2	39	47	
3	8	12	
Acute Rejection	86	129	0.96+
Type of CNI; Cyclosporin: Tacrolimus	290∶15	434∶21	0.90+
Anti-CD25 monoclonal antibody	50	82	0.59+
HLA mismatch (no. of antigens)			
0	44	58	0.62+
1	31	41	
2	128	206	
3	71	108	
4	15	25	
5	10	14	
6	4	5	
**Mean mismatches per patient**	**2.09**	**2.15**	
DR locus mismatch (no. of antigens)			
0	207	297	0.42+
1	83	142	
2	13	18	
**Mean mismatches per patient**	**0.36**	**0.41**	

As the analysis involved the comparison between absence of HLA-C2 in the recipient versus presence of HLA-C2 in the recipient groups were divided accordingly. Absence of HLA-C2 represents HLA-C1 homozygous whereas presence of HLA-C2 combines both heterozygous and HLA-C2 homozygous groups. CI is 95% confidence interval.

*indicates significance by ANOVA test,

+ indicates significance by Kendall's tau-b test.

**Table 4:** Cox regression multivariable analysis for all factors that may influence outcomes after kidney transplantation.

	Significance	Hazard ratio	95% CI	Significance	Hazard ratio	95% CI	Significance	Hazard ratio	95% CI
	Graft survival, death non-censored			Graft survival, death censored			Patient survival		
Recipient age	0.29	0.99	0.98–1.01	**0.002**	**0.98**	**0.96–0.99**	**0.001**	**1.05**	**1.02–1.08**
Donor age	0.04	1.01	1.00–1.03	0.02	1.02	1.00–1.03	0.69	1.01	0.98–1.03
Recipient sex	0.77	1.05	0.75–1.47	0.50	1.20	0.71–2.05	0.88	0.95	0.49–1.85
Donor sex	0.61	1.09	0.79–1.51	0.52	1.14	0.77–1.67	0.19	0.63	0.32–1.25
Seropositive CMV in recipient	0.18	0.78	0.55–1.12	0.19	0.76	0.51–1.15	0.71	0.87	0.41–1.84
Seropositive CMV in donor	0.48	0.89	0.63–1.24	0.41	0.85	0.57–1.25	0.84	0.93	0.46–1.87
Type of transplant: Deceased versus Live	**0.02**	**2.96**	1.18–7.44	0.11	2.17	0.85–5.54	0.77	1.05	0.76–1.48
Number of transplant									
**Comparisons made to 1^st^ transplant**									
2	0.83	0.94	0.55–1.62	0.17	0.61	0.30–1.24	0.11	2.10	0.86–5.14
3	0.49	1.52	0.46–5.03	0.34	1.80	0.54–6.08	0.99	0.00	0.00
Acute Rejection	0.35	1.17	0.84–1.65	0.15	1.33	0.91–1.95	0.38	0.70	0.32–1.55
Cyclosporin versus Tacrolimus	0.35	0.64	0.25–1.65	0.57	1.53	0.35–6.63	**0.003**	**0.15**	**0.04–0.51**
Anti-CD25 monoclonal antibody	0.62	1.15	0.66–1.98	0.89	1.04	0.58–1.89	0.36	2.03	0.45–9.13
HLA mismatch (no. of antigens)									
**Comparisons made to 0 HLA mismatch**									
1	0.07	1.90	0.96–3.75	0.05	2.13	0.99–4.61	0.76	1.28	0.27–5.96
2	0.29	1.37	0.77–2.45	0.40	1.34	0.68–2.63	0.70	1.25	0.40–3.87
3	0.86	0.94	0.46–1.94	0.61	0.80	0.33–1.90	0.83	1.16	0.31–4.36
4	**0.04**	**2.46**	**1.05–5.75**	0.05	2.60	1.00–6.75	0.78	1.40	0.14–14.28
5	0.63	1.56	0.25–9.66	0.28	2.69	0.45–16.03	0.98	0.00	0.00
6	0.43	3.01	0.20–45.16	0.23	6.67	0.30–149.09	0.99	0.00	0.00
DR locus mismatch (no. of antigens)									
**Comparisons made to 0 DR mismatch**									
1	0.56	1.15	0.73–1.80	0.47	1.22	0.71–2.08	0.99	1.01	0.42–2.38
2	0.50	0.55	0.09–3.17	0.25	0.26	0.03–2.55	0.18	8.53	0.39–188.23
HLA-C2 absent in recipient	**0.007**	**1.55**	**1.13–2.13**	**0.02**	**1.53**	**1.06–2.21**	0.19	1.54	0.80–2.94
HLA-Bw4 absent in recipient	0.35	1.17	0.84–1.65	0.50	1.20	0.71–2.05	0.88	0.95	0.49–1.85

Statistically significant associations are indicated in bold.

In table 5 the causes of graft loss were analysed in the context of the absence or presence of HLA-C2. This analysis demonstrated that chronic allograft injury and vascular thrombosis occurred more frequently in the absence of HLA-C2 when compared to the presence of HLA-C2.

**Table 5:** Causes of graft loss categorized by the absence or presence of HLA-C2.

Cause of graft loss	Absence of HLA-C2(n = 303)	Presence of HLA-C2(n = 457)	P value
Chronic allograft injury	60 (20%)	64(14%)	0.03
Vascular thrombosis	16 (5.3%)	8 (1.7%)	0.01
Acute rejection	2 (0.7%)	5 (1.1%)	NS
Recurrent of primary renal disease	2 (0.7%)	4 (1.1%)	NS
Infection of allograft	1 (0.3%)	1 (0.3%)	NS

Figures reported are absolute number of events and figures in brackets represent percentages calculated for events per HLA-C genotype. Statistical analysis was performed by Kendall's tau-b test. NS represents not significant.

### HLA-C and KIR genotypes do not influence the incidence of acute rejection

The correlation between the incidence of biopsy proven acute rejection (33% in our kidney transplant population) and various genotypes for donor and recipient was studied. Complete data was available for 657 cases. The remaining 103 cases were excluded because these patients were referred to other treatment centre for their routine transplant follow-up and accurate data regarding acute rejection episodes were not available. The incidence of acute rejection did not correlate with individual KIR genotype, KIR haplotype, KIR ligand genotype (HLA-C or Bw4), or KIR ligand matching.

### Donor derived NK cells are present in the allograft at the time of transplant

**Figure 4:**
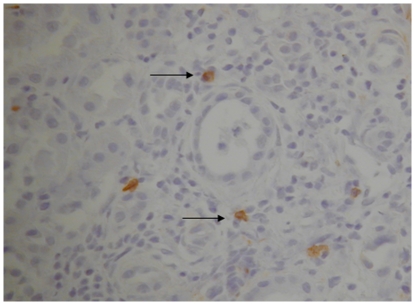
Immunohistochemistry slide demonstrating donor derived CD56 positive cells (anti-CD56 antibody staining brown in colour: arrow as indicator) in pre-transplant kidney biopsy tissue. In brief, the method for development of this slide included dewaxing and antigen retrieval obtained by W-cap system (Bio-Optica) and staining with mouse monoclonal anti-CD56 antibody (IgG2b Novocastra) used at a dilution of 1∶50 and visualised with the EnVision detection system (DAKO). This image is representative of the observation made for biopsies taken from five different kidney transplants studied. Cell counts (degree of infiltration) were performed using light microscopy and counting 10 randomly selected high power fields at a magnification of 400× (area = 0.17 mm^2^). Mean (±SEM) of 3±2 CD56 positive cells were identified per high power field.

Immunohistochemical staining for CD56 confirms the presence of NK cells in pre-transplant kidney tissue providing evidence that at transplantation donor derived NK cells are present in the allograft (figure 4). Cell counts (degree of infiltration) were performed using light microscopy and counting 10 randomly selected high power fields at a magnification of 400× (area = 0.17 mm^2^). Mean (±SEM) of 3±2 CD56 positive cells were identified per high power field.

### Optimal DC maturation in allogeneic NK-DC co-culture requires cytokine IL-15 activation and is cell contact dependent

Allogeneic interactions between NK and iDC without cytokine activation led to minimal DC maturation. This was consistent with previous observations [23], [26]. In co-culture, the addition of 1 ng/ml of IL-15 significantly augmented DC maturation with increased expression of CD86, HLA-DR and CCR7. DC maturation occurred optimally at NK-DC ratios of 1∶1 consistent with previous reports [21], [22]. In trans-well experiments where IL-15 activated NK and DC were separated by a porous membrane, DC maturation did not occur, confirming that IL-15 activated NK cell mediated DC maturation is contact dependent.

### HLA-C2 expression by DC is associated with inhibited maturation responses in NK-DC co-culture

**Figure 5:**
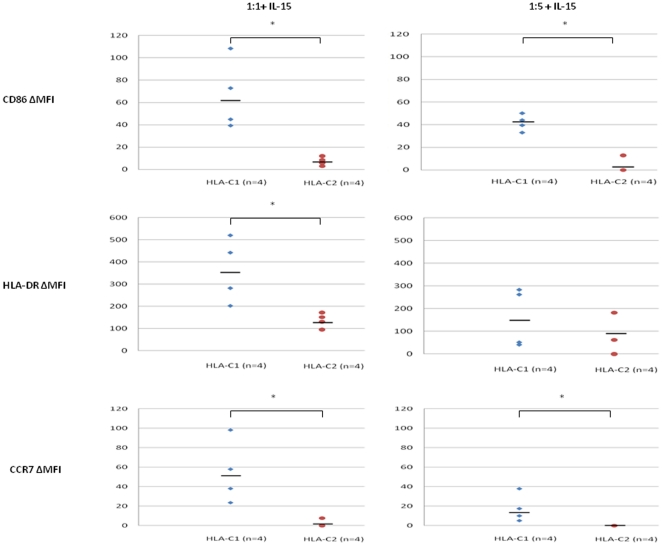
Comparisons are made for ΔMFI of CD86, HLA-DR and CCR7 expression between DCs with either HLA-C1 or HLA-C2 homozygous allele. Data shown are ΔMFI of CD86, HLA-DR and CCR7 expressed by DC in NK-DC co-culture in the presence of 1 ng/ml IL-15 at cell ratios of either 1∶1 or 1∶5. ΔMFI are calculated as the difference of MFI for DC in co-culture versus DC in isolation i.e. spontaneous expression. In NK-DC co-culture, in the presence of IL-15, DC with HLA-C1 homozygous allele express more co-stimulation molecules, and MHC class II molecules than DC with HLA-C2 homozygous alleles. Furthermore expression of trafficking chemokine CCR7 is virtually exclusive to HLA-C1 homozygotes indicating their predominant role in T-cell immune priming in secondary lymphoid tissues. Data shown for 4 independent experiments performed in each group and * indicates statistical significance with p<0.05 by Mann Whitney U test.

**Figure 6:**
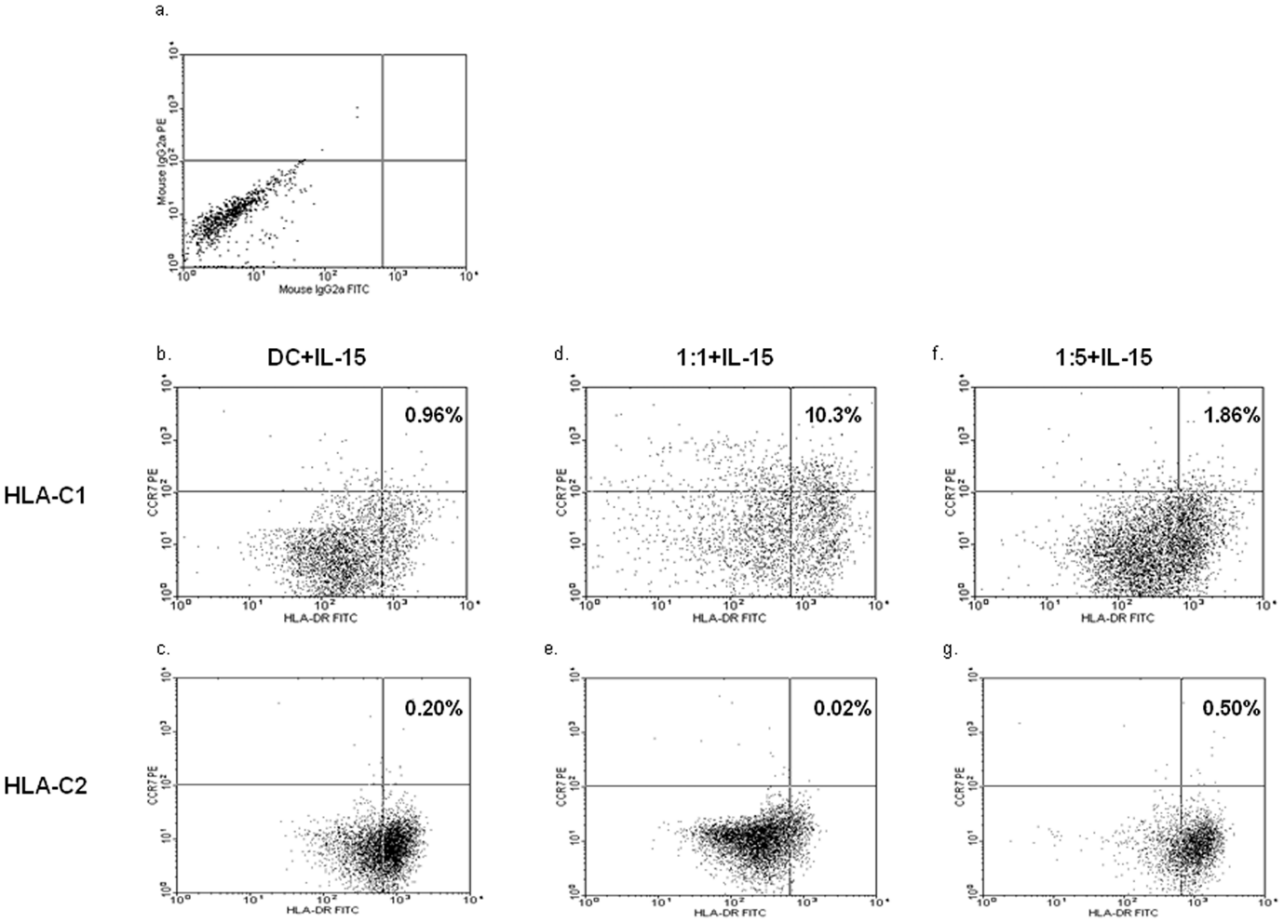
Flow cytometer data illustrating the impact of IL-15 (1 ng/ml) treated NK-DC co-culture on the expression of DC maturation (HLA-DR FITC) and chemokine (CCR7 PE) markers, comparing responses for DCs with either HLA-C1 (**figures 6b, d, f**) or HLA-C2 (**figures 6c, e, g**) homozygous alleles. (a) shows DC stained with isotype control for HLA-DR & CCR7, (b) & (c) shows background DC staining where DCs are in isolation in the presence of IL-15, (d) & (e) shows HLA-DR & CCR7 expression by DC in NK-DC co-culture at ratios of 1∶1 in the presence of IL-15, (f) & (g) shows DC markers in NK-DC co-culture at ratios 1∶5 in the presence of IL-15. This data clearly demonstrates that in NK-DC co-culture at ratios of 1∶1 in the presence of IL-15, HLA-C1 homozygous DCs undergo significantly greater maturation than HLA-C2 homozygous DCs and attain CCR7 chemokine expression required for trafficking to secondary lymphoid tissues. Results are representative of 4 experiments with 5,000 DC gated events captured.

The influence of HLA-C genotype on DC maturation in NK-DC co-culture was assessed at cell ratios of 1∶1 and 1∶5 in the presence of 1 ng/ml IL-15. In NK-DC co-culture at 1∶1 cell ratios, DC maturation (CD86 and HLA-DR) and chemokine expression (CCR7) were significantly greater for HLA-C1 DCs (ΔMFI: CD86 = 61.08±15.40, HLA-DR = 363.67±73.21, CCR7 = 52.35±16.23) compared to HLA-C2 DCs (ΔMFI: CD86 = 6.43±6.12, HLA-DR = 115.81±40.00, CCR7 = 1.91±1.91,) (p<0.05, Mann-Whitney U test) (figure 5 & 6). Similarly at NK-DC cell ratios of 1∶5, CD86 and CCR7 expression were significantly greater for HLA-C1 DCs (ΔMFI: CD86 = 44.78±10.39, CCR7 = 14.35±8.77) than HLA-C2 DCs (ΔMFI: CD86 = 3.22±3.22, CCR7 = 0.00±0.00, p<0.05, Mann-Whitney U test) (figure 5 & 6). However, at 1∶5 cell ratios HLA-DR expression was not significantly different in HLA-C1 DCs compared with HLA-C2 DCs (159.97±64.82 vs 94.42±39.97, p = NS). In general, CD86 and HLA-DR expression by DCs were greater at cell ratios of 1∶1 than 1∶5. In co-culture, CCR7 expression occurred mainly on HLA-C1 DCs expressing alleles (figure 5 & 6). CCR7 expression was more marked at NK-DC co-culture cell ratios of 1∶1 (10.3% versus 1.86%) (figure 6).

### HLA-C2 expression by DC is associated with an anti-inflammatory cytokine milieu in NK-DC co-culture

**Figure 7:**
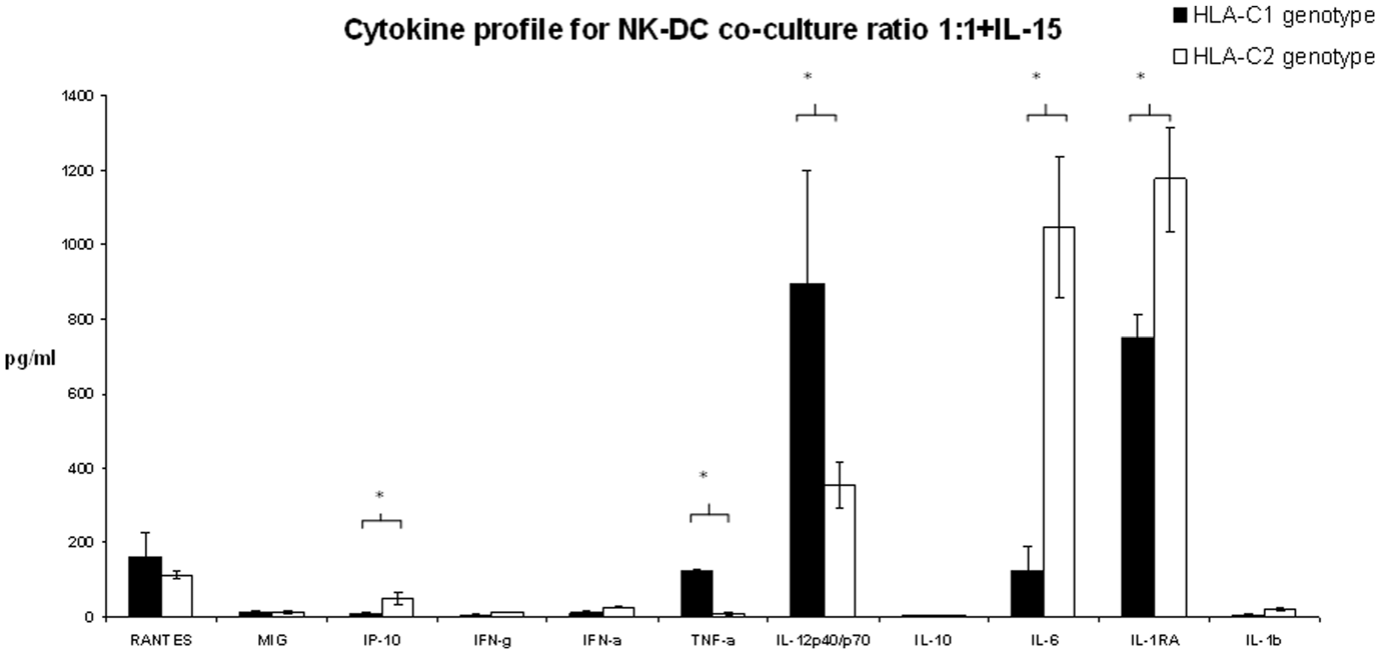
Supernatants were collected from NK-DC co-culture experiments at cell ratios of 1∶1 treated with IL-15 and tested for cytokine synthesis using 25 cytokine multiplex bead immunoassay. Data shown are for all the cytokines that were expressed in culture supernatants. Of these, comparisons were made between cytokine synthesis for DCs with either HLA-C1 homozygous allele or DCs with HLA-C2 homozygous alleles. Data shown excludes background synthesis by cells in isolation. These data indicates that DC with HLA-C1 alleles express significantly greater pro-inflammatory cytokines in NK-DC co-culture favouring immune maturation whereas DC with HLA-C2 alleles predominantly express anti-inflammatory cytokines that are inhibiting to DC maturation. Means ± SEM for 4 independent experiments in each group are shown and * indicates statistical significance with p<0.05 by Mann-Whitney U test.

**Figure 8:**
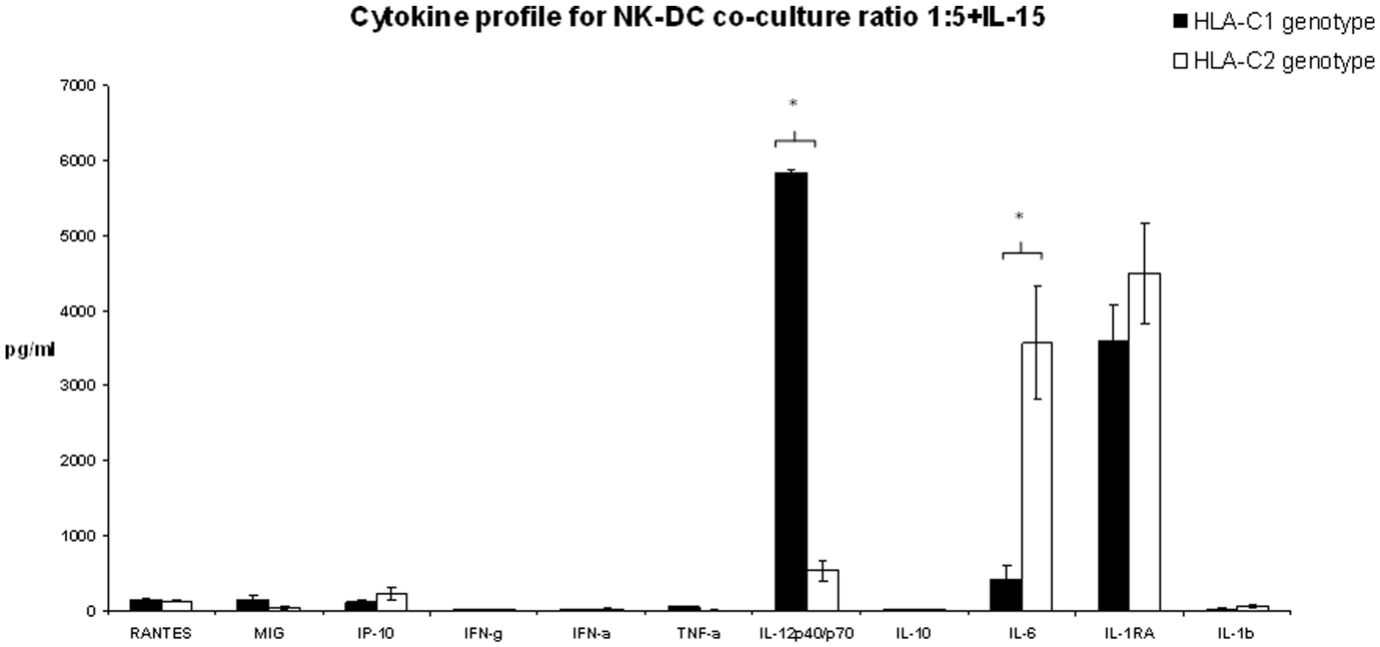
Supernatants were collected from NK-DC co-culture experiments at cell ratios of 1∶5 treated with IL-15 and tested for cytokine synthesis using 25 cytokine multiplex bead immunoassay. Data shown are for all the cytokines that were expressed in culture supernatants. Of these, comparisons were made between cytokine synthesis for DCs with either HLA-C1 homozygous allele or DCs with HLA-C2 homozygous alleles. Data shown excludes background synthesis by cells in isolation. These data indicates that DC with HLA-C1 alleles express significantly greater pro-inflammatory cytokines in NK-DC co-culture favouring immune maturation whereas DC with HLA-C2 alleles predominantly express anti-inflammatory cytokines that are inhibiting to DC maturation. Means ± SEM for 4 independent experiments in each group are shown and * indicates statistical significance with p<0.05 by Mann-Whitney U test.

Supernatants were collected from NK-DC co-culture and tested for cytokine synthesis using 25 cytokine multiplex bead immunoassay. From the cytokines analysed, 11 could be detected (as shown on figures 7 and 8). Differential cytokine synthesis was compared between HLA-C1 DC and HLA-C2 DC in co-culture in the presence of 1 ng/ml IL-15. Data presented in figure 7 and 8 are means ± SEM for 4 independent experiments in each arm (HLA-C1 versus HLA-C2) and takes into account background cytokine production when DC/NK cells were in isolation. In NK-DC co-culture at cell ratios of 1∶1 (figure 7), significantly more TNF-α and IL-12 were detected in supernatants from HLA-C1 DC (TNF-α: 123.51±5.1 pg/ml; IL-12: 895.91±302.73 pg/ml) compared to HLA-C2 DC (TNF-α: 10.42±3.02 pg/ml; IL-12: 354.71±64.23 pg/ml, p<0.05). In contrast, significantly more IP-10, IL-6 and IL-1RA were detected in supernatants from HLA-C2 DC (IP-10: 49.43±15.22 pg/ml; IL-6: 1046.16±191.54 pg/ml; IL-1RA: 1175.03±142.25 pg/ml) than HLA-C1 DC (IP-10: 10.92±1.34 pg/ml; IL-6: 126.25±66.48 pg/ml; IL-1RA: 752.11±58.94 pg/ml, p<0.05). Other cytokines including RANTES, MIG, interferon-γ, interferon-α, IL-10 and IL-1β were detected in comparable quantities between groups. In NK-DC co-culture at cell ratios of 1∶5 (figure 8), significantly more IL-12 was detected in supernatants from HLA-C1 DC (IL-12: 5825.28±54.34 pg/ml) compared with HLA-C2 DC (IL-12: 533.99±129.51 pg/ml, p<0.05). In contrast, significantly more IL-6 was detected in supernatants from HLA-C2 DC (IL-6: 3564.48±750.53 pg/ml) than HLA-C1 DC (IL-6: 424.07±179.14 pg/ml, p<0.05). Other cytokines including RANTES, MIG, interferon-γ, interferon-α, TNF-α, IL-10, IL-1RA and IL-1β were detected at comparable quantities between the groups. TNF-α and IL-12 are potent inducers of DC maturation whereas IL-6 and IL-1RA have been shown to inhibit DC maturation. This differential cytokine profile is consistent with the observation that, in co-culture with NK cells, HLA-C1 DC expressed more CD86, HLA-DR and CCR7 than HLA-C2 DC. Diminished TNF-α synthesis during co-culture at cell ratios of 1∶5 (figure 8) may explain the lower expression of CD86, HLA-DR and CCR7 when compared to 1∶1 cell ratios (figure 7).

## Discussion

In contrast to liver transplantation, recipient expression of a HLA-C2 allele had a strong impact on clinical outcomes following kidney transplantation. We found that kidney transplant recipients with a HLA-C2 allele had a 21.9% death non-censored and 19.9% death censored better graft survival at 10 years when compared to recipients without the allele. Recipients with either a HLA-C2 homozygous or heterozygous allele had similar graft survival benefits, suggesting that gene dose does not have a strong effect in this setting. The relative risk of graft loss for kidney transplant recipients without a HLA-C2 allele was 1.5 times that of recipients with a HLA-C2 allele. The benefit of a HLA-C2 allele in the recipient was further confirmed by multivariable analysis. Re-transplants were included because analysing re-transplant cases alone demonstrated similar survival benefits; this observation is fundamentally important as it further emphasizes the reliability of this data, through a recipient derived effect that is maintained even in re-transplantation. 10-year patient survival was also superior in the HLA-C2 positive recipients. However this effect was not significant in the multivariable analysis; this indicates any associations of recipient HLA-C2 allele with patient survival independent of graft loss will be weak. People with kidney transplant failure are treated with dialysis and therefore rarely die from graft failure. Causes of graft loss were analysed in the context of the absence or presence of HLA-C2; this analysis demonstrated that the absence of HLA-C2 was associated with more chronic allograft injury and vascular thrombosis.

Whilst HLA mismatching is known to influence graft survival our study was not designed to detect these as the number of cases per HLA mismatch were small. Other observations made on the multivariable analysis including a lack of a consistent benefit seen with HLA matching, variable effects seen for increasing recipient age with graft and patient survival, and the beneficial effect on patient survival in the tacrolimus group must be interpreted with caution as this study was not designed to investigate these parameters. In particular patients from our clinical centre who were started on tacrolimus were generally younger patients which may have had a direct impact on survival outcomes.

We also tested the impact of the ‘missing self’ model on kidney transplant outcomes as previously performed by Tran and colleagues [18]. Consistent with their observations, we found no correlation between KIR ligand mismatch and graft survival. HLA-C ligand matching also did not influence the rate of acute rejection.

Kunert and colleagues [16] studied the relationship between the incidence of acute rejection after kidney transplantation and various KIR genotypes for a cohort of 224 patients. They found a reduced incidence of acute rejection when the donor was HLA-C2 homozygous. In contrast with their work, we found no significant association between a donor HLA-C2 homozygous genotype and acute rejection. More broadly, we tested both donor and recipient genotypes and found no association between HLA-C ligands, KIR or KIR haplotypes and acute rejection in 657 kidney transplant patients. The explanation for the conflicting findings may relate to their small study size (HLA-C2 homozygous, n = 31, p = 0.052), and reflect a type I statistical error.

The organ specific directional benefit of possession of HLA-C2 allele (recipients with a HLA-C2 allele in kidney transplants and donors with HLA-C2 allele in liver transplants [15]) is of great interest. These observations are consistent with other data that liver and kidney transplants develop immunological responses through different mechanisms [36], [37].

In experimental models of transplantation, liver allografts have been shown to be immune privileged, with evidence for tolerance against T cells [38]–[40]. The mechanisms described include induction of CD8^+^ T-cell apoptosis [38], inhibition of CD4^+^ T-cell mediated Th1 differentiation [39] and production of the anti-inflammatory cytokine IL-10 [40]. In clinical transplantation, liver allografts are transplanted without HLA matching and prior knowledge of anti-donor HLA antibodies. HLA matching is not associated with improvement in long-term graft survival after liver transplantation [41]. Therefore current evidence indicates that chronic rejection may be attributable to recipient NK cells targeting donor tissue [15], [37], [42]–[45]. Based on our hypothesis, an absence of HLA-C2 allele on the allograft would lead to NK cell activation with consequent liver transplant damage [15]. Conversely kidney transplants are highly susceptible to all components of the adaptive immune system. CAI occurs through adaptive immune processes involving T cells, B cells and anti-HLA antibodies [46]. Therefore the associations between the recipient HLA-C2 allele and survival benefit may be linked to the maturation of adaptive immunity facilitated by interactions between donor-derived NK cells and recipient antigen presenting cells (stimuli for indirect allorecognition) in the early post-transplant period. Indeed, there is good evidence linking indirect allorecognition to poor long-term graft function in kidney transplantation [47]–[49].

The increased understanding of NK cell biology in the past decade has been based on studies that have assessed interactions between NK cells and DC. DC are professional antigen presenting cells with very potent T-cell priming ability [50]. Recently, activated NK cells were shown to interact with DC to efficiently induce their maturation [51]–[53]. Subsequently, Brilot and colleagues [27] showed polarisation of KIR and KIR ligands to the site of cell-cell contact in NK-DC co-culture indicating that this interaction is involved in NK-DC cross talk. Vitale and colleagues [26] showed that the predominant activation signal in NK mediated DC maturation occurs with engagement of the natural cytotoxic receptor NKp30. This signal was modulated by blocking antibodies against a HLA specific inhibitory receptor (A6/136 IgM), indicating the involvement of KIRs and KIR ligands. Therefore, at the NK-DC immune synapse, HLA-C2 expression by DC may inhibit DC maturation following engagement with KIR 2DL1, with a consequent impact on priming of the adaptive immune system.

Potent NK-DC interactions require a favourable cytokine milieu [51], and one important cytokine in this setting is IL-15 [27]. Kidney tissue constitutively expresses IL-15 [31], [33], with up-regulated expression in the early stages post-transplantation [32], [54]. Unlike IL-2, IL-15 synthesis is not abrogated by anti-CD25 monoclonal antibody treatment [55]. Furthermore, IL-15 induced activation of NK cells is not inhibited by conventional induction immunosuppression with methylprednisolone [56] and encourages potent NK-DC interactions.

We developed an allogeneic NK-DC co-culture model which is relevant to the setting of kidney transplantation. In support of the validity of this model, immunohistochemical studies performed on pre-transplant kidney tissue confirms that donor derived NK cells are present in the allograft at the time of the transplant. Following transplantation, with up-regulation of constitutively expressed IL-15 [31], [33], [54] donor NK cells interact with immature DC. We demonstrated that on in-vitro allogeneic NK-DC co-culture, significant DC maturation occurred following treatment with 1 ng/ml of IL-15 and this process was cell contact dependent. DC maturation occurred at NK-DC cell ratios of either 1∶1 or 1∶5, but not when NK cells were in excess of DC at a ratio of, 5∶1. NK-DC cell ratios of 1∶1 were maximally inductive for DC maturation. This observation is very relevant to the clinical setting, as donor derived cells may be at lower numbers than recipient cells following allograft reperfusion.

We made the novel observation that in NK-DC co-culture, the HLA-C allele expressed by DC influenced the degree of DC maturation. In co-culture, HLA-C1 DC (without HLA-C2 allele) underwent more maturation with greater expression of CD86 (a co-stimulation molecule) and HLA-DR (a MHC class II molecules) when compared to HLA-C2 DC. Furthermore, CCR7, a chemokine receptor required for cell trafficking to secondary lymphoid tissue [57] was exclusively expressed by HLA-C1 DC. CCR7 expression has been shown to enhance DC migratory speed [58], [59] and survival [60]. Therefore HLA-C1 DC (without HLA-C2 allele), matured in-situ in the days following kidney transplantation, acquire the capacity to migrate to secondary lymphoid tissue, where they will present antigen to promote the activation and proliferation of T cells specific for graft antigen [61]. Conversely, DC with HLA-C2 allele express low level CD86 and HLA-DR with no CCR7 expression. This may make them inefficient at T-cell priming but may provide them with tolerogenic potential [62], [63].

Supernatants from NK-DC co-culture were also assessed for the presence of cytokines. At cell ratios of 1∶1 in presence of 1 ng/ml of IL-15, TNF-α and IL-12 were detected at significantly higher concentrations for HLA-C1 DC than HLA-C2 DC. Conversely, IP-10, IL-6 and IL-1 receptor antagonist (IL-1RA) were detected at higher concentrations with HLA-C2 DC than with HLA-C1 DC. These cytokine profiles are consistent with the observation of enhanced cell surface maturation markers for HLA-C1 DC and a failure of efficient maturation for HLA-C2 DC in co-culture. TNF-α and IL-12 are pro-inflammatory cytokines involved in DC maturation [21]–[23]. IL-12 synthesised by DC in NK-DC co-culture has been shown to augment the activation status of NK cells [64]. This in-turn may favour further IFN-γ, TNF-α, and GM-CSF, encouraging DC maturation [65]. The cytokine profiles noted for HLA-C2 DC are anti-inflammatory; the increased IP-10 synthesis may reflect the prolonged contact of ineffective DCs with NK cells. This may be relevant in the process of DC editing. Furthermore, IL-6 inhibits NF-κB binding activity and CCR7 expression [66], therefore inhibiting DC maturation. IL-1 is a powerful pro-inflammatory cytokine profoundly inhibited by IL-1 receptor antagonist (IL-1RA). The role of IL-1RA in human diseases is well described [67]. Up-regulation of IL-1RA may therefore impede DC maturation in HLA-C2 DCs.

Based on these observations, recipients without HLA-C2 alleles are at greater risk of accelerated DC maturation compared with recipients with HLA-C2 alleles. As a consequence recipients without a HLA-C2 allele are subjected to a greater immunological load through T-cells primed by indirect allorecognition.

In conclusion, early events in kidney transplantation involving donor NK cells and recipient DC interactions via KIR and HLA-C immune synapse has a major impact on subsequent immune priming; this is determined by the recipient HLA-C genotype. Graft survival outcomes are related to these processes, which therefore represent a prime target for therapeutic intervention to prolong allograft survival. Furthermore, recipients with the HLA-C2 allele may be at lower immunological risk and may therefore benefit from the minimisation of immunosuppression.
